# Renal function is a major predictor of circulating acyl-CoA-binding protein/diazepam-binding inhibitor

**DOI:** 10.3389/fendo.2023.1152444

**Published:** 2023-05-23

**Authors:** Robin Schürfeld, Benjamin Sandner, Annett Hoffmann, Nora Klöting, Ekaterine Baratashvili, Marcin Nowicki, Sabine Paeschke, Joanna Kosacka, Susan Kralisch, Anette Bachmann, Armin Frille, Anja Dietel, Jens-Uwe Stolzenburg, Matthias Blüher, Ming-Zhi Zhang, Raymond C. Harris, Berend Isermann, Michael Stumvoll, Anke Tönjes, Thomas Ebert

**Affiliations:** ^1^ Medical Department III – Endocrinology, Nephrology, Rheumatology, University of Leipzig Medical Center, Leipzig, Germany; ^2^ Department of General, Visceral, Transplant, Vascular and Pediatric Surgery, University Hospital Würzburg, Würzburg, Germany; ^3^ Helmholtz Institute for Metabolic, Obesity and Vascular Research of the Helmholtz Zentrum München at the University of Leipzig and University Hospital, Leipzig, Germany; ^4^ Department of Cardiology, Angiology and Internal Intensive-Care Medicine, Klinikum St. Georg, Leipzig, Germany; ^5^ Institute of Anatomy, University of Leipzig, Leipzig, Germany; ^6^ Department of Visceral, Transplant, Thoracic and Vascular Surgery, University of Leipzig Medical Center, Leipzig, Germany; ^7^ Department of Respiratory Medicine, University Hospital Leipzig, University of Leipzig, Leipzig, Germany; ^8^ Department of Urology, University of Leipzig, Leipzig, Germany; ^9^ Division of Nephrology, Department of Medicine, Vanderbilt University School of Medicine, Nashville, TN, United States; ^10^ Department of Medicine, Nashville Veterans Affairs Hospital, Vanderbilt University School of Medicine, Nashville, TN, United States; ^11^ Institute of Laboratory Medicine, Clinical Chemistry, and Molecular Diagnostics, University Hospital Leipzig, Leipzig, Germany

**Keywords:** Acyl-CoA-binding protein, adipokines, chronic kidney disease, diabetic kidney disease, diazepam binding inhibitor, hemodialysis, type 2 diabetes mellitus

## Abstract

**Objective:**

Acyl-CoA-binding protein (ACBP)/diazepam-binding inhibitor has lately been described as an endocrine factor affecting food intake and lipid metabolism. ACBP is dysregulated in catabolic/malnutrition states like sepsis or systemic inflammation. However, regulation of ACBP has not been investigated in conditions with impaired kidney function, so far.

**Design/methods:**

Serum ACBP concentrations were investigated by enzyme-linked immunosorbent assay i) in a cohort of 60 individuals with kidney failure (KF) on chronic haemodialysis and compared to 60 individuals with a preserved kidney function; and ii) in a human model of acute kidney dysfunction (AKD). In addition, *mACBP* mRNA expression was assessed in two CKD mouse models and in two distinct groups of non-CKD mice. Further, mRNA expression of *mACBP* was measured *in vitro* in isolated, differentiated mouse adipocytes - brown and white - after exposure to the uremic agent indoxyl sulfate.

**Results:**

Median [interquartile range] serum ACBP was almost 20-fold increased in KF (514.0 [339.3] µg/l) compared to subjects without KF (26.1 [39.1] µg/l) (p<0.001). eGFR was the most important, inverse predictor of circulating ACBP in multivariate analysis (standardized β=-0.839; p<0.001). Furthermore, AKD increased ACBP concentrations almost 3-fold (p<0.001). Increased ACBP levels were not caused by augmented *mACBP* mRNA expression in different tissues of CKD mice *in vivo* or in indoxyl sulfate-treated adipocytes *in vitro*.

**Conclusions:**

Circulating ACBP inversely associates with renal function, most likely through renal retention of the cytokine. Future studies need to investigate ACBP physiology in malnutrition-related disease states, such as CKD, and to adjust for markers of renal function.

## Introduction

Chronic kidney disease (CKD) has a leading global health impact and is a multisystem disease state accounting for about 1.2 million deaths per year worldwide (1). Key pathomechanisms include a proinflammatory status, increased oxidative stress and cellular senescence (2, 3), thereby contributing to increased mortality with about 40-50% of deaths attributed to cardiovascular diseases (4). Cardiometabolic disease states frequently observed in patients with CKD and kidney failure (KF) include diabetes (5, 6), dyslipidemia (7) and a proinflammatory phenotype. Therefore, potential endocrine cytokines linking cardiometabolic diseases with impaired renal function need to be investigated.

Recently, acyl-CoA binding protein (ACBP), also called diazepam binding inhibitor, has been introduced as an endocrine factor affecting food intake and lipid metabolism (8). ACBP is ubiquitously expressed throughout the body with highest levels observed in human liver, brain, kidney and gut (9). Bravo-San Pedro and co-workers demonstrated that intravenous injection of recombinant ACBP in mice enhances food intake, while lowering glucose levels. Furthermore, it activates lateral hypothalamic orexigenic neurons and increases lipogenic gene expression, e.g. fatty acid synthase in hepatocytes and white adipocytes (8). Conversely, ACBP-neutralizing antibodies induced hyperglycemia, hypophagia, and weight loss in mice (8). In accordance, a systemic knockout of the ACBP gene results in less weight gain following a high-fat diet in adult mice (10) and are more sensitive to fasting-induced weight loss (8). ACBP has also been reported to positively correlate with BMI in human studies supporting its role as an obesogenic factor (8, 11, 12). Importantly, conditions of severe catabolic states like sepsis or systemic inflammation are correlated to increased plasma ACBP levels (13, 14). As malnutrition and sarcopenia frequently occur in patients with advanced CKD and KF (2), the regulation of circulating ACBP in patients with CKD and KF needs to be investigated in more detail. Therefore, we quantified plasma ACBP concentrations in 60 individuals with KF on maintenance haemodialysis (HD) treatment and compared it to 60 individuals with an estimated glomerular filtration rate (eGFR) > 50 ml/min/1.73m². In addition, we measured ACBP in 20 patients with acute kidney dysfunction (AKD) after unilateral nephrectomy. Furthermore, we examined *mACBP* mRNA expression in two CKD mouse models due to diabetic kidney disease (i.e. *eNOS^-/-^*;*db/db* mice and *db/db* mice on a C57BLKS/J background) in relation to two mouse models without CKD (i.e. heterozygous *db/+* mice and eNOS^-/-^ mice). Finally, we determined mRNA expression of *mACBP* in cultured, differentiated murine brown and white fat cells after exposure to the uremic toxin indoxyl sulfate versus control treatment. Our main hypothesis was that ACBP is eliminated by the kidneys and hence ACBP is increased in i) CKD patients with HD treatment and ii) in patients with AKD.

## Materials and methods

### Subjects

#### Study population 1 (CKD)

For the investigation of ACBP regulation in human CKD, a cross-sectional study previously described in (15, 16) was used. The following inclusion criteria were required to participate in the study: age >18 years, non-pregnant, provided written informed consent. Patients were excluded if they had end-stage malignancy, acute generalized inflammation, acute infectious disease or a history of drug abuse. For the current analysis, 120 Caucasian participants (women: N = 58; men: N = 62) were available. Of the total cohort (N=120), about 60 (50%) patients had a kidney failure (KF) and were on maintenance HD. In study population 1, blood samples were obtained after an overnight fast of at least 8 hours. In all KF patients, blood draw was executed immediately before the start of HD. In a subgroup of 10 patients with KF (5 with type 2 diabetes (T2D) and 5 without T2D), serum ACBP concentrations were measured before and immediately after HD. Study participants with a preserved eGFR > 50 ml/min/1.73m² (N = 60) defined by the Chronic Kidney Disease Epidemiology Collaboration (CKD-EPI) formula (17) were used as control subjects. In all women and men, fasting glucose (FG) ≥ 7.0 mmol/l or use of insulin or other glucose-lowering medication was classified as type 2 diabetes (T2D) (18). Based on these criteria, 32 of the 60 patients with KF on maintenance HD and 30 out of 60 control patients were classified as T2D. The study was approved by the Ethics Committee of the University of Leipzig (Reg. No: 180-13-15072013), and written informed consent was obtained from all volunteers prior to their participation in the study.

#### Study population 2 (AKD)

To investigate ACBP in a human model of AKD, a second study population of patients undergoing elective partial or total unilateral nephrectomy was used, as previously described (19, 20). Former studies already established that these patients serve as a reliable model for AKD (21, 22). Inclusion criteria were an age between 18 and 80 years and provided written informed consent, whereas patients were excluded for the following criteria: presence of hemodialysis therapy, hereditary renal cysts, glomerulonephritis, and generalized inflammation. In brief, 61 consecutively recruited patients (17 women, 44 men) received elective partial or total unilateral nephrectomy at the Department of Urology of Leipzig University Hospital. In all patients, fasting blood samples were drawn before, as well as within 30 hours after, renal surgery. For the present analysis, 20 subjects were available. The study was approved by the Ethics Committee of the University of Leipzig (Reg. No: 029-12-23012012), and written informed consent was obtained from all volunteers prior to their participation in the study.

### Biochemical analysis/Clinical parameters

Serum levels of ACBP (DBI Human enzyme-linked immunosorbent assay (ELISA) Kit, #KA0532, Abnova, Taipeh, Taiwan) as well as high sensitivity interleukin 6 (hsIL6, #HS600B, R&D Systems, Wiesbaden, Germany) were measured using an ELISA according to the manufacturers’ instructions. Routine serum parameters, including creatinine, FG, fasting insulin (FI), triglycerides (TG), high density lipoprotein (HDL), low density lipoprotein (LDL) cholesterol, and C-reactive protein (CRP) were measured in a certified laboratory by standard methods. Using FG and FI, homeostasis model assessment of insulin resistance (HOMA2-IR) was determined as previously described (23).

### Animal study

To investigate tissue-specific ACBP regulation in CKD, two mice models of CKD caused by diabetic kidney disease (DKD) (*eNOS^-/-^;db/db* mice (i.e. mice with severe DKD (24) and *db/db* mice (i.e. mice with mild DKD (25)) were compared to two non-diabetic control groups (non-diabetic *eNOS^-/-^* mice, as well as *db/+* mice (26)). All stated mice were on a C57BLKS/J background. All mice were kept under pathogen-free conditions at 21 ± 1°C on a 12-hour light/dark cycle (6 AM to 6 PM) and received free access to water and a normal chow pellet diet. At 24 weeks of age, mice were sacrificed by exsanguination while maintainingdeep anesthesia with intravenous ketamine (WDT, Garbsen, Germany) and xylazine (Bayer Health Care, Wuppertal, Germany). For later RNA isolation, biopsies of liver, kidney, visceral (VAT), subcutaneous (SAT), and brown (BAT) adipose tissue, as well as hypothalamus, were shock frozen in liquid nitrogen. Location of all animal experiments was the Medical Experimental Center at the University of Leipzig with previously described methods (26–28). The local ethics committee of the state of Saxony (Landesdirektion Leipzig) permitted all animal experiments (approval nos. TVV 12/14 and TVV 65/15).

### *mACBP* mRNA expression in murine brown and white adipocytes during incubation with the uremic toxin indoxyl sulfate *in vitro*

Immortalized murine brown preadipocytes were cultured according to (29) In brief, selected preadipocytes were grown to confluence in culture medium supplemented with differentiation medium. After confluence, cells were incubated in differentiation medium supplemented with induction medium. Subsequently, the cells were maintained only in differentiation medium for 4–5 days until exhibiting a fully differentiated phenotype. Murine 3T3-L1 white preadipocytes (American Type Culture Collection, Rockville, MD) were also grown to confluence in culture medium. Confluent cells were then differentiated to adipocytes by supplementation of culture medium with insulin, isobutylmethylxanthine, and dexamethasone as outlined in (30). Using the methods of Stockler-Pinto et al. (31), as well as Gondouin et al. (32), after starvation for a period of 24 hours, brown and white adipocytes were incubated with 1 mM indoxyl sulfate for 24 hours. For controls, cells were also exposed for 24 hours to 1 mM potassium sulfate (Sigma, Darmstadt, Germany) according to the recommendations of the European Uremic Toxin Work Group (EUTox) (33). Both, indoxyl sulfate and potassium sulfate, were diluted in NaCl 0.9%.

### Analysis of *mACBP* mRNA expression

*mACBP* mRNA expression was determined relative to acidic ribosomal phosphoprotein P0 (*m36B4*) using quantitative real-time RT-PCR. This includes all animal and *in vitro-*experiments. In more detail, 1 µg total RNA was obtained from each sample and reverse transcribed in a 20 µl reverse transcription reaction. 1 µl of each reverse transcription reaction, producing cDNA, was used on a LightCycler 480 real-time PCR 96 well thermocycler using LightCycler 480 Probes Master Mix (Roche Diagnostics GmbH, Mannheim, Germany) essentially as described in (26, 34). Primer pairs with the following sequence were used: *mACBP*, TTTCGGCATCCGTATCACCT (sense) and TTTGTCAAATTCAGCCTGAGACA (antisense); *m36B4*, AAGCGCGTCCTGGCATTGTCT (sense) and CCGCAGGGGCAGCAGTGGT (antisense).

### Statistical analyses

SPSS software version 27.0 (IBM, Armonk, NY) and GraphPad Prism 9 (GraphPad Software Inc., La Jolla, CA) were used for all statistical analyses. In study population 1, overall group differences between the four subgroups were assessed by non-parametric Kruskal-Wallis test with Bonferroni *post hoc* analysis for continuous parameters, whereas χ²-test was used for categorical parameters. To conduct univariate correlation analyses, we used Spearman’s rank correlation methods. To identify independent associations between ACBP and cardiometabolic and anthropometric markers, linear regression analysis was calculated. For this purpose, parameters that correlated significantly with ACBP in univariate analysis (except for covariates) were included in the multivariate model with further adjustment for sex. Prior to carrying out multivariate linear regression analysis, all non-normally distributed parameters were logarithmically transformed.

For analysis of paired samples in study population 1 (i.e. before vs. after hemodialysis in a subset) and in study population 2 (i.e. before vs. after unilateral nephrectomy), Wilcoxon signed-rank test was used.

In the animal and *in vitro* experiments, relative *mACBP* mRNA expression in different mouse tissues and adipocytes were analyzed by one-way ANOVA with Bonferroni *post hoc* tests, as well as by Student’s t test, after logarithmical transformation prior the analyses.

A p-value of <0.05 was considered as statistically significant in all analyses.

## Results

### Baseline characteristics of study population 1 (CKD, N = 120)

**Figure 1:**
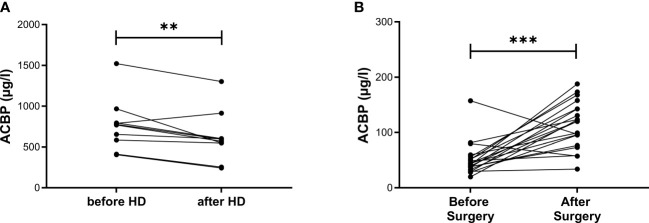
ACBP serum concentrations before and after HD as well as before and after unilateral nephrectomy in matched samples of patients. ACBP serum concentrations were measured before and immediately after hemodialysis (HD) in 10 subjects from study population 1 in **(A)** and before, as well as within 30 hours after, partial or total unilateral nephrectomy in 20 subjects from study population 2 in **(B)**. Each data point refers to one patient. P-values were calculated by Wilcoxon signed-rank test. ** indicates p < 0.01, ***p < 0.001.

Study population 1 was subdivided into the four described subgroups (i.e. Control/nonT2D; Control/T2D; HD/nonT2D; HD/T2D). Basic clinical characteristics of each group are displayed in **Table 1**. Median [interquartile range] serum ACBP levels were 109.7 [488.6] µg/l in the total sample and did not depend on sex (p = 0.472) and T2D (p = 0.610). In contrast, patients with KF (514.0 [339.3] µg/l) had significantly, almost 20-fold, higher serum concentrations of ACBP as compared to subjects with a preserved renal function (26.1 [39.1] µg/l) (p < 0.001). In the subgroup of 10 patients with ACBP levels before and after HD, serum ACBP level were decreased from 772.7 [297.1] (before HD) to 581.4 [205.6] µg/l (after HD) (**Figure 1A**, p < 0.01).

**Table 1:** Baseline characteristics of the study population.

	Subgroup 1	Subgroup 2	Subgroup 3	Subgroup 4	Overall p
	Control/nonT2D	Control/T2D	HD/nonT2D	HD/T2D	
N	30	30	28	32	–
Sex (male/female)	11/19	16/14	15/13	20/12	0.239
ACBP (µg/l)	42.9 (46.0) ^§,#^	23.1 (11.7) ^§,#^	510.0 (351.0) ^*,†^	523.2 (324.3) ^*,†^	<0.001
Age (years)	63 (19)	63 (16)	59 (23)	68 (12)	0.051
BMI (kg/m²)	28.2 (5.6)	29.1 (5.2) ^§^	25.2 (6.5) ^†^	27.9 (6.6)	0.004
Waist circumference (cm)	94.8 (20.9) ^#^	102.8 (19.8)	99.3 (24.6)^#^	117.8 (20.0) ^*,§^	0.002
SBP (mmHg)	125 (21)	126 (20)	125 (38)	120 (25)	0.339
DBP (mmHg)	77 (10)	73 (15)	77 (20)	70 (18)	0.095
FG (mmol/l)	5.1 (1.3) ^†^	7.6 (3.2) ^*§,#^	4.6 (1.2) ^†^	5.2 (3.3) ^†^	<0.001
FI (pmol/l)	45.1 (33.3)	47.9 (62.6)	28.2 (47.6)	50.1 (91.6)	0.23
HOMA2-IR	0.85 (0.60)	1.10 (1.32)	0.55 (0.88)	0.90 (1.63)	0.101
Cholesterol (mmol/l)	5.3 (0.9) ^§,#^	4.9 (1.5)	4.4 (1.1) ^*^	4.2 (1.3) ^*^	<0.001
HDL cholesterol (mmol/l)	1.4 (0.4) ^§,#^	1.2 (0.5) ^#^	1.0 (0.5) ^*^	1.0 (0.3) ^*,†^	<0.001
Non-HDL cholesterol (mmol/l)	4.2 (1.0) ^§,#^	3.5 (1.3)	3.2 (1.2) ^*^	3.1 (1.2) ^*^	0.001
LDL cholesterol (mmol/l)	3.5 (1.1) ^†,§,#^	2.9 (0.9) ^*^	2.7 (0.9) ^*^	2.1 (1.4) ^*^	<0.001
TG (mmol/l)	1.1 (0.8) ^§,#^	1.4 (0.9)	1.6 (0.9) ^*^	1.8 (1.4) ^*^	<0.001
Creatinine (μmol/l)	76 (17) ^§,#^	72 (22) ^§,#^	829 (431) ^*,†^	717 (221) ^*,†^	<0.001
eGFR (ml/min/1.73m^2^)	78.8 (24.4) ^§,#^	85.2 (23.0) ^§,#^	5.0 (3.2) ^*,†^	5.7 (2.7) ^*,†^	<0.001
hsIL-6 (ng/l)	1.87 (2.11) ^§,#^	2.07 (1.25) ^§,#^	6.14 (4.91) ^*,†^	7.48 (7.30) ^*,†^	<0.001
CRP (mg/l)	2.5 (4.4)	2.8 (4.6)^#^	3.5 (11.9)	6.9 (24.7)^†^	0.024

Baseline characteristics of the study population stratified by the four subgroups, i.e. Control/nonT2D, Control/T2D, HD/nonT2D, and HD/T2D. ACBP, Acyl-CoA binding protein; BMI, Body mass index; DBP, Diastolic blood pressure; eGFR, Estimated glomerular filtration rate; FFA, Free fatty acids; FI, Fasting insulin; HD, Hemodialysis; HDL, High density lipoprotein; HOMA2-IR, Homeostasis model assessment of insulin resistance; hsIL-6, High sensitivity interleukin-6; LDL, Low density lipoprotein; SBP, Systolic blood pressure; T2D, Type 2 diabetes; TG, Triglycerides; CRP, C-reactive protein. Values for median (interquartile range) or total numbers are shown. Categorical parameters were analyzed using the χ²-test. Continuous parameters were analyzed by Kruskal-Wallis test followed by Bonferroni-adjusted post hoc analysis. Overall p values of the Kruskal-Wallis test are depicted. Superscript numbers (^*,†,§,#^) indicate subgroup comparisons with significant Bonferroni-adjusted post hoc analyses. ^*,†,§,#^ indicate p<0.05 as compared to Subgroup 1 (^*^) (control/nonT2D), Subgroup 2 (^†^) (control/T2D), Subgroup 3 (^§^) (HD/nonT2D), and Subgroup 4 (^#^) (HD/T2D), respectively.

When patients were stratified by the four subgroups, i.e. Control/nonT2D; Control/T2D; HD/nonT2D; HD/T2D, circulating ACBP differed significantly between the four subgroups (p < 0.001) and the HD subgroups had significantly higher ACBP concentrations compared to both non-HD subgroups (**Table 1**).

### Correlation of ACBP with anthropometric and biochemical parameters in study population 1 (CKD, N = 120)

Analysing all 120 individuals, ACBP serum levels were positively correlated to age, WHR, TG, creatinine, hsIL-6, and CRP (all p < 0.05; **Table 2**). Furthermore, ACBP was negatively related to BMI, FG, cholesterol, HDL cholesterol, non-HDL cholesterol, LDL cholesterol, and eGFR (all p < 0.05; **Table 2**).

**Table 2:** Univariate correlation analyses and multivariate linear regression analysis of serum ACBP with anthropometric parameters and markers of glucose metabolism, serum lipids, inflammation, and renal function.

	Univariate correlation analyses		Multivariate linear regression analysis	
	r	p	β	p
Age (years)	0.181	0.047	0.033	0.363
Sex	N/A	N/A	-0.160	0.681
BMI (kg/m^2^)	-0.201	0.028	-0.008	0.825
Waist circumference (cm)	–	n.s.	–	–
SBP (mmHg)	–	n.s.	–	–
DBP (mmHg)	–	n.s.	–	–
FG (mmol/l)	-0.362	<0.001	-0.069	0.074
FI (pmol/l)	–	n.s.	–	–
HOMA2-IR	–	n.s.	–	–
Cholesterol (mmol/l)	-0.309	0.001	–	–
HDL cholesterol (mmol/l)	-0.341	<0.001	0.010	0.820
Non-HDL cholesterol (mmol/l)	-0.238	0.009	–	–
LDL cholesterol (mmol/l)	-0.356	<0.001	0.019	0.640
TG (mmol/l)	0.242	0.008	–	–
Creatinine (µmol/l)	0.829	<0.001	–	–
eGFR (ml/min/1.73m²)	-0.851	<0.001	-0.839	<0.001
hsIL-6 (ng/l)	0.667	<0.001	0.106	0.030
CRP (mg/l)	0.202	0.027	–	–

Univariate correlation analyses and multivariate linear regression analysis of ACBP with anthropometric and biochemical markers. Non-parametric Spearman’s rank correlation method was used to assess univariate relationships between ACBP and indicated markers. Multivariate regression analysis was calculated for ACBP (lg, dependent variable) adjusted for age (lg), sex, BMI (lg), FG (lg), HDL cholesterol (lg), LDL cholesterol (lg), eGFR (lg), as well as hsIL-6 (lg). Non-normally distributed variables as assessed by Shapiro-Wilk-test were logarithmically transformed prior to multivariate testing (lg). r- and p-values, as well as standardized β-coefficients and p-values, are given. Abbreviations are indicated in **Table 1**. N/A, not applicable. n.s., not statistically significant.

### Correlation of ACBP with metabolic parameters in study population 1 (CKD, N = 120)

Again, analysing all 120 individuals, eGFR was the most relevant, significant, and negative predictor of ACBP levels (p < 0.001; **Table 2**) after adjustment for age, sex, and cardiometabolic markers in linear regression analysis. Furthermore, hsIL-6 positively associated with circulating ACBP (p = 0.030; **Table 2**). On the other hand, ACBP was not independently associated with markers of obesity, glucose homeostasis, and dyslipidemia in multivariate linear regression analysis in the entire cohort (**Table 2**) nor with CRP when included instead of hsIL-6 (**Supplementary Table S1**, Model 2).

### Study population 2 (AKD)

Baseline characteristics, as well as pre- and postsurgery biochemical parameters, have already been described elsewhere (28). Median serum concentrations of ACBP were significantly, almost 3-fold, higher after surgery (120.0 [52.1] µg/l) compared to before surgery (43.2 [17.1] µg/l) (**Figure 1B**, p < 0.001).

### *mACBP* mRNA expression in DKD mice

**Figure 2:**
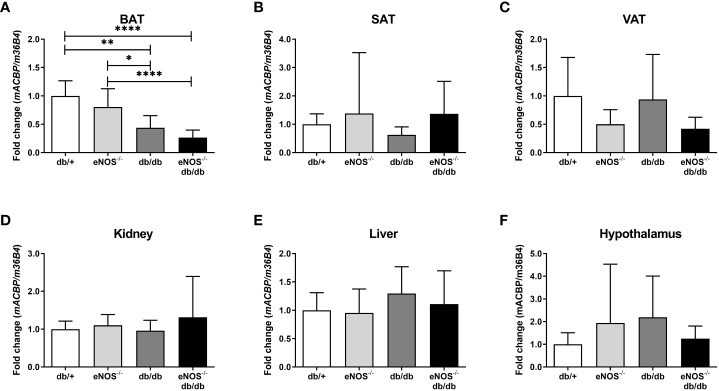
*mACBP* mRNA expression in a mouse model of diabetic kidney disease compared to control mice. In all experiments, mice with 24 weeks of age with severe diabetic kidney disease (DKD) (*eNOS^-/-^; db/db*; black bars) were compared to obese, diabetic (*db/db*; dark grey bars), lean, endothelial dysfunctional (*eNOS^-/-^* mice; light grey bars), as well as healthy, lean control mice (*db/+* mice; white bars). **(A–F)** *mACBP* mRNA expression normalized to *m36B4* in **(A)** brown adipose tissue (BAT), **(B)** subcutaneous adipose tissue (SAT), **(C)** visceral adipose tissue (VAT), **(D)** kidney, **(E)** liver, and **(F)** hypothalamus. Results are displayed as means ± standard deviation. p-values were calculated by one-way ANOVA including Bonferroni adjustment for post-hoc tests. N ≥ 5 per group. *indicates p < 0.05, **p < 0.01, ****p<0.0001 for subgroup comparisons.

**Figure 2** depicts tissue-specific *mACBP* mRNA expression in insulin-sensitive tissues (i.e. liver and adipose tissue depots), as well as kidney and hypothalamus, in four groups of mice with different renal function, i.e. healthy, lean *db/+* mice vs. endothelial dysfunctional *eNOS^-/-^* mice vs. obese, diabetic *db/db* mice with mild DKD vs. obese, diabetic *eNOS^-/-^;db/db* mice with severe DKD.

In BAT, *mACBP* mRNA expression was significantly decreased in 24-week-old CKD mice. We detected thelowest *mACBP* expression in mice with severe DKD, i.e. *eNOS^-/-^;db/db* (**Figure 2A**, overall p < 0.001). In contrast, *mACBP* mRNA expression did not differ between the animal groups in SAT, VAT, kidney, liver, and hypothalamus (**Figures 2B–F**, all p > 0.05).

### *mACBP* mRNA expression levels in murine 3T3-L1 adipocytes *in vitro* after incubation with the uremic toxin indoxyl sulfate

**Figure 3:**
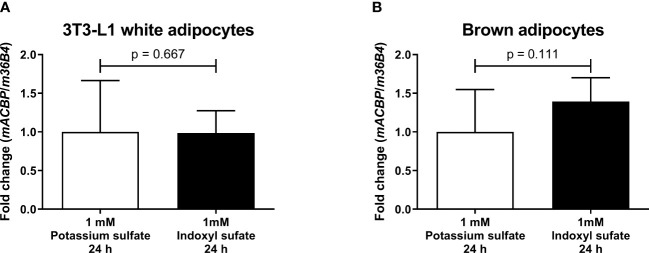
*mACBP* mRNA expression after treatment in 3T3-L1 white adipocytes and brown adipocytes with uremic toxin indoxyl sulfate. *mACBP* mRNA expression in **(A)** murine 3T3-L1 white adipocytes, and **(B)** differentiated, immortalized murine brown adipocytes after 24 hours-lasting incubation with 1 mM indoxyl sulfate and control cells exposed to 1 mM potassium sulfate for 24 hours. The *mACBP* mRNA expression is shown as normalized to *m36B4*. Results are displayed as means ± standard deviation. p-values were calculated by Student’s t test. N ≥ 5 per group.

To investigate whether uremic toxins contribute to an altered *mACBP* mRNA expression in adipocytes *in vitro*, murine 3T3-L1 white adipocytes, as well as brown adipocytes, were treated with the major uremic toxin indoxyl sulfate for 24 hours compared to potassium sulfate. Mean *mACBP* mRNA expression was non-significantly altered after indoxyl sulfate treatment compared to control cells in 3T3-L1 white adipocytes (**Figure 3A**, p = 0.667) and brown adipocytes (**Figure 3B**, p = 0.111).

## Discussion

Here we show, that ACBP is significantly, almost 20-fold, increased in patients with KF receiving HD treatment as compared to control subjects, and almost 3-fold increased in patients with AKD after unilateral nephrectomy compared to presurgical concentrations. Furthermore, eGFR, as well as hsIL-6, are independently associated with circulating ACBP concentrations in patients with KF.

Similar to ACBP, several other cytokines with cardiometabolic effects are also significantly elevated in KF, including leptin (35), adiponectin (36), adipocyte fatty acid-binding protein (19), and follistatin-like 3 (28). To investigate whether increased ACBP levels in CKD could be due to enhanced ACBP protein synthesis in various tissues, we analyzed *mACBP* mRNA expression in two CKD mouse models as compared to two distinct non-diabetic control mouse models. *mACBP* mRNA expression is downregulated in brown adipocytes in CKD mice, whereas gene expression of the cytokine in other tissues is not different between CKD and non-CKD mice. Furthermore, mRNA expression of *mACBP* is not modified by the uremic toxin indoxyl sulfate in adipocytes *in vitro.* Based on these results, increased ACBP serum levels in KF are not caused by augmented mRNA expression of the cytokine in the uremic milieu (i.e. similar to adiponectin (36)), but rather by decreased kidney function leading to subsequent retention of ACBP. In agreement with this rationale and with the findings in KF, ACBP levels are increased after unilateral nephrectomy in a human AKD model, as well. While HD treatment can partially remove ACBP from the circulation (**Figure 1A**), ACBP concentrations remain to be significantly elevated after HD treatment compared to controls indicating only a small effect of HD on ACBP levels. Our results appear to be independent of the cause of KF, and ACBP concentrations do not differ in KF due to DKD vs. other diseases. It should be noted that ACBP has been negatively related to eGFR in the French general population-based DESIR study, in a French advanced cancer cohort and in 63 randomly selected patients undergoing a short-term weight loss program (12, 37). Importantly, mean eGFR was 85.3 ml/min/1.73m² in the DESIR study (12), and therefore, these cohorts (12, 37), are not applicable to investigate ACBP regulation in patients with CKD in more detail.

Interestingly, ACBP is not independently associated with BMI in multivariate analysis in our cohort, while several other studies have shown a positive association of BMI and ACBP (8, 11, 12, 37, 38). However, patients from all of these studies have not been specifically selected for CKD, and in several cohorts even been excluded in the presence of advanced CKD/KF (8, 11, 12, 37, 38). Furthermore, the association of ACBP with BMI is lost in cancer patients and patients with cardiovascular diseases (12), and cancer patients with malnutrition even show an inverse correlation of ACBP with BMI (37). Malnutrition and sarcopenia are also common in CKD (2) and hence may contribute to increased ACBP levels, hypothetically partly through autophagy, which causes ACBP release to extracellular space (8, 39). It is, therefore tempting to speculate that in healthier conditions ACBP levels are positively related to BMI, whereas this relationship is lost or even reversed in individuals with CVD and malnutrition-related diseases, e.g. cancer and KF. Future studies, therefore, need to elucidate whether impaired ACBP regulation in these disease states is causally involved in malnutrition and impaired body composition status, thereby contributing to CVD.

Apart from markers of renal function, CRP and hsIL-6 are positively correlated with ACBP levels in the univariate analysis of study population 1, with hsIL-6 furthermore being independently correlated in the multivariate analysis. This is probably due to the higher sensitivity of IL-6 for chronic inflammation. Interestingly, ACBP’s cleavage peptide ODN enhances LPS-stimulated IL-6 secretion of human monocytes (40), as well as ROS production in human neutrophils (41), *in vitro*. Furthermore, ACBP levels are increased in a rat model of sepsis (13) and conversely, ACBP-neutralizing antibodies inhibit proinflammatory pathways and profibrotic genes, while upregulating antioxidant resources in mice (42, 43). This implicates that ACBP is involved in pro-inflammatory pathways, but future studies need to determine whether the cytokine contributes to systemic inflammation or is driven by it.

In the univariate correlation analyses, ACBP was associated with a dyslipidemic profile. These results are in line with a meta-analysis by Joseph et al. demonstrating a positive association of ACBP levels with TG (37). Furthermore, Montegut and co-workers have validated these findings observing not only a positive correlation between ACBP and TG, but also a negative correlation between HDL cholesterol and ACBP, independent of age and BMI (12), supporting our findings in patients with impaired renal function.

Some limitation of the current study need to be pointed out. First, ACBP is known to be associated with short- and long-term caloric ingestion (37), so correlation of caloric intake with ACBP levels would be informative, but was lacking in our cohort. Second, possible pre- to post surgery differences, for instance increased CRP after surgery and/or a prolonged fasting state (37) during the perioperative period, could have influenced ACBP levels in study population 2. Third, only mRNA expression levels for *mACBP* were used to account for possible changes in ACBP serum levels, while protein expression data either by western blot or by immunohistochemistry could reflect circulating ACBP more accurate. Last, the number of animals used in our experiments may have been too low to identify potential differences in mRNA expression of *mACBP*.

In conclusion, ACBP plasma concentrations are almost 20-fold higher in patients with KF on HD treatment as compared to control subjects and almost 3-fold higher in a human model of AKD. As *mACBP* mRNA expression is not increased in the uremic milieu, circulating ACBP is most likely increased in CKD due to retention. Therefore, markers of renal function need to be reported in future studies on ACBP. Future studies should focus on the interplay of malnutrition, inflammation and impaired renal function, which all influence ACBP concentrations and are to be encountered in CKD patients. Whether an anti-ACBP therapy may reduce inflammation, remains to be investigated.

## Data availability statement

The raw data supporting the conclusions of this article will be made available by the authors, without undue reservation.

## Ethics statement

The studies involving human participants were reviewed and approved by Ethics Committee of the University of Leipzig. The patients/participants provided their written informed consent to participate in this study. The animal study was reviewed and approved by Local ethics committee of the state of Saxony (Landesdirektion Leipzig).

## Author contributions

RS, BS, AT, and TE wrote the manuscript and researched data. AH, NK, EB, MN, SP, SK and AB researched data and reviewed/edited the manuscript. MB, M-ZZ, RCH, BI, AF, AD, J-US, and MS contributed to the discussion and reviewed/edited the manuscript. Guarantor: RS and TE are the guarantors of this work and, as such, had full access to all the data in the study and take responsibility for the integrity of the data and the accuracy of the data analysis. All authors contributed to the article and approved the submitted version.
