# Supplementary material for: Renal function is a major predictor of circulating acyl-CoA-binding protein/diazepam-binding inhibitor
Source: Front Endocrinol (Lausanne). 2023 May 23;14:1152444. doi: 10.3389/fendo.2023.1152444 (PMC10242139; doi:10.3389/fendo.2023.1152444)
Supplement: Supplementary file 1 [file Table_1.docx]

| **Supplementary Table S1:**  Multivariate linear regression analysis of serum ACBP with anthropometric parameters  and markers of glucose metabolism, serum lipids, inflammation, and renal function in study population 1. | | | | |
| --- | --- | --- | --- | --- |
|  | **Model 1** | | **Model 2** | |
|  | **β** | **p** | **β** | **p** |
| Age (years) | **0.078** | **0.030** | 0.039 | 0.289 |
| Sex | 0.070 | 0.069 | -0.015 | 0.704 |
| BMI (kg/m^2^) | -0.013 | 0.725 | -0.006 | 0.877 |
| FG (mmol/l) | -0.063 | 0.103 | -0.068 | 0.081 |
| HDL cholesterol (mmol/l) | 0.010 | 0.812 | 0.011 | 0.809 |
| LDL cholesterol (mmol/l) | 0.015 | 0.700 | 0.016 | 0.702 |
| Creatinine (µmol/l) | **0.852** | **<0.001** | **-** | **-** |
| eGFR (ml/min/1.73m²) | - | **-** | **-0.898** | **<0.001** |
| hsIL-6 (ng/l) | **0.104** | **0.032** | **-** | **-** |
| CRP (mg/l) | - | - | 0.047 | 0.242 |

Different multivariate linear regression analyses models for serum ACBP (lg, dependent variable) with anthropometric and biochemical markers in study population 1. Calculations were adjusted for age (lg), sex, BMI (lg), FG (lg), HDL cholesterol (lg) and LDL cholesterol (lg) in all models. Creatinine (lg) and hs-IL-6 (lg) were further used for adjustments in analysis 1. eGFR (lg) and CRP (mg/l) were further used for adjustments in analysis 2. Non-normally distributed variables as assessed by Shapiro-Wilk-test were logarithmically transformed prior to multivariate testing (lg). Standardized β-coefficients and p-values are given. Abbreviations are indicated in Table 1.
